# Profiling crystal engineered ligands for targeting treatment resistant androgen receptors

**DOI:** 10.3389/fphar.2026.1813002

**Published:** 2026-09-28

**Authors:** Avan Navaz Colah, Charles Izuchukwu Ezekiel, Sára Ferková, Pierre-Luc Boudreault, Leonard R MacGillivray, William A Ricke

**Affiliations:** 1 Division of Pharmaceutical Sciences, University of Wisconsin-Madison School of Pharmacy, Madison, WI, United States; 2 Department of Chemistry, University of Iowa, Iowa, IA, United States; 3 Département de Pharmacologie-physiologie, Faculté de Médecine et des Sciences de La Santé, Université de Sherbrooke, Sherbrooke, QC, Canada; 4 Département de Chimie, Faculté des Sciences, Université de Sherbrooke, Sherbrooke, QC, Canada; 5 Department of Urology, University of Wisconsin School of Medicine and Public Health, Madison, WI, United States

**Keywords:** androgen receptor point mutation, castration resistant prostate cancer, crystal engineering, molecular docking, solid state synthesis

## Abstract

**Introduction:**

Prostate cancer (PCa) is one of the principal contributors to health burden in the aging male population. PCa develops through dysregulation of androgen receptor (AR) signaling pathways. Despite improvements in diagnostic techniques and interventions, no pharmacological measures with long term efficacy have been established once PCa advances to castration resistant prostate cancer (CRPC). To circumvent this issue, tetra-aryl cyclobutanes (CBs) have been proposed as structurally distinct compounds with a mechanism of action differing from traditional androgen receptor pathway inhibitor (ARPI).

**Methods:**

Here, we apply principles of crystal engineering and solid state synthesis to expand the class of CBs through strategic derivatization. The synthesis of the CB occurs quantitatively, producing no side products. We demonstrate how head-to-tail stacking interactions of halo-pyrimidine rings can be exploited to stack and align unsymmetrical alkenes to undergo [2 + 2] photodimerization to generate the CB in the solid state. We examine the structure-function relationships of CBs in vitro by profiling AR mediated transcriptional activity, receptor translocation, and cell viability. Moreover, we explored putative binding interactions of ClCB and BrCB ligands across distinct AR mutants (wild-type, T877A, and F877L/T878A) using molecular docking.

**Results/Discussion:**

In total, our data suggest that CBs have unexploited therapeutic potential in CRPC and that green chemistry and crystal engineering principles offer a unique route to generate these drug candidates.

## 1 Introduction

Prostate cancer (PCa) is the second most frequently diagnosed malignancy in men worldwide and is primarily driven by dysregulation to the androgen receptor signaling pathway (Culig and Santer, 2014). As PCa progresses to castration resistant prostate cancer (CRPC), most cases remain androgen receptor (AR) positive despite mechanistic deviation in AR signaling. Sequencing of advanced stage prostate cancers revealed that 20% of genomic alterations to AR were attributed to AR point mutation (Beltran et al., 2013). These point mutations to the AR ligand binding domain (LBD) arise in response to the selective pressure of androgen receptor pathway inhibitor (ARPI) therapy, and result in increased receptor promiscuity and a shift in recognition of ARPIs as AR agonists rather than AR antagonists (Steketee et al., 2002; Veldscholte et al., 1990). Examples of therapeutic shift have been reported post treatment with both first- and second-generation ARPIs including the hydroxyflutamide (Flut) induced T878A mutation (Veldscholte et al., 1990), the bicalutamide induced W742C mutation (Hara et al., 2003), and the enzalutamide (Enza) and apalutamide induced F877L mutation (Korpal et al., 2013; Joseph et al., 2013; Gim et al., 2021). Due to the emergence of the F877L point mutation and the increase in Enza resistant CRPC, darolutamide (Daro) was developed to inhibit AR LBDs with the F877L point mutation (Borgmann et al., 2018). Within patient populations, the prevalence of co-occurrent point mutations to AR is increasing in response to consecutive rounds of treatment with ARPIs after initial treatment failure (Ledet et al., 2020). Although new small molecules like Daro can be rationally designed for clinical use, the vast majority of AR LBD targeting therapeutic bind to the LBD with a highly similar mechanism, making them susceptible to resistance point mutations (Liu et al., 2015; Liu et al., 2016; Liu et al., 2017a; Liu et al., 2017b). Moreover, the increased potency of second-generation ARPIs favors the emergence of resistance mechanisms since there is strong selective pressure for cell survival. Therefore, there is a critical need for a broad-spectrum AR antagonist that can inhibit a range of AR mutants consistently and with long term efficacy, now and into the future.

Recent reports have documented cyclobutane (CB) molecules as prospective drug candidates (Van Der Kolk et al., 2022). CBs are experiencing an upsurge of interest in a broad range of areas related to synthetic chemistry and materials science (Lee-Ruff and Mladenova, 2003). CBs are naturally formed through UV-induced intermolecular [2 + 2] photocycloadditions (Xu et al., 2015). The central CB ring acts as a core to emanate pendant substituents, such as aryl groups, rigidly situated in space (Carreira and Fessard, 2014). Whereas solution phase approaches to CB ring formation typically result in CB mixtures, the use of crystal engineering principles to direct formations of CBs has become highly desirable to improve product formation. CBs can now often be generated in the crystalline state as single products and in near 100% yield, thus eliminating needs for compound purification (Sinnwell et al., 2021). Moreover, the use of the solid state to form CBs of therapeutic value can be considered an unexpected, unexplored, and attractive avenue for pharmaceutical development (Sinnwell et al., 2021). Generating cyclobutane photoproducts as AR antagonists is an exciting prospect given the remarkable ease of the solid state reactivity and the potential to form CBs that are inaccessible in solution-based approaches. This is particularly timely, as feasible methods for generating structurally distinct therapeutic candidates are increasingly important when successfully bringing drug candidates to market.

The current understanding of CBs as point mutated AR antagonists in CRPC can be attributed to a report exploring a tetra-aryl cyclobutane scaffold as a base for drug design (Pollock et al., 2016). From this work, we chose the CB scaffold with -Cl substituents—which we define as the ClCB—as the lead compound with potential in CRPC (Pollock et al., 2016). The rigidity and unique structure of ClCB confers a distinct mechanism of AR inhibition, whereby AR/CB complexes are retained in the cytoplasm and maintain an unliganded-like receptor conformation (Pollock et al., 2016). Despite promising capacities, such CBs have yet to progress to clinical trials. Therefore, complementary work building upon these initial mechanistic findings is necessary to foster development of CBs as therapeutic candidates in CRPC. Our primary goal is to better understand the potential of the ClCB and other halogenated CBs including the newly developed bromide -Br derivatives (BrCB) as drug candidates in CRPC and further establish the CB scaffold as a foundational structure for rational drug design. We demonstrate scientific advancement through our application of solid-state synthesis to compound derivatization, and using structural modeling to evaluate putative binding interactions between AR/CB complexes. Moreover, we define adaptive ligand binding as potential mechanistic rationale for the mutational resilience of CBs observed in cellular models of CRPC.

## 2 Materials and methods

### 2.1 Synthesis of (E)-2-bromo-4-styrylpyrimidine (BrSP)

The synthesis of the BrSP follows the procedures of the coupling reaction as reported (Tan et al., 2004). *Trans*-2-phenylvinylboronic acid (0.8226 g, 1 eq, 5.6 mmol) was dissolved in tetrahydrofuran (25 mL). PdCl_2_(PPh_3_)_2_ (117 mg, 0.03 eq, 0.17 mmol) was added to the solution followed by potassium phosphate tribasic (2.377 g, 2 eq, 5.6 mmol). 2,4-dibromopyrimidine (1.332 g, 1 eq, 5.6 mmol) was then dissolved in THF (5 mL) and added to the stirred solution and then followed by water (5 mL). The reaction flask was equipped with a condenser and brought to reflux for 24 h. The reaction was quenched with water (25 mL). The organic products were extracted with ethyl acetate (3 × 25 mL). The resulting solutions were combined and washed with brine (30 mL), dried over Na_2_SO_4_, and concentrated under reduced pressure. The resulting solid was purified through recrystallization using 10% ethyl acetate in hexanes to afford pure BrSP (0.8062 g, 61%). Solid-state irradiation of BrSP. Finely ground samples of BrSP (200 mg) were immersed between two Pyrex glass plates and irradiated using a 500 W broadband mercury lamp. ^1^H NMR spectroscopy was used to monitor the progress of the photoreaction, and near 100% conversion was achieved in a period of 12 h. The photoproduct was purified through recrystallization using 10% ethyl acetate in hexanes to provide pure solid BrCB in a near quantitative recovery. A similar procedure was used to synthesize and irradiate (E)-2-chloro-4-styrylpyrimidine (ClSP) to generate ClCB.

Acquisition of crystal structures. About 10 mg of each of ClSP and ClCB were dissolved in ethanol and allowed to slowly evaporate. Plate-like crystals suitable for SCXRD were obtained after 24 h. Similarly, about 10 mg each of BrSP and BrCB were dissolved in benzene and acetonitrile (1:1) and allowed to slowly evaporate. Plate-like crystal suitable for SCXRD was obtained after 48 h. Mercury 4.2.0 (RRID:SCR_004231) was used to generate representations of crystal structures, stacking, intermolecular interactions, and packing.

### 2.2 Cell culture reagents and media conditions

CB compounds were prepared as stock solutions in DMSO. The ARPIs Enzalutamide (MDV3100, Astellas) and Darolutamide (ODM-201, MedChem Express) were solubilized in DMSO (>98% purity). The synthetic androgen R1881 (AbMole) was prepared in DMSO. LNCaP/AR, LNCaP (RRID:CVCL_0395), and MR49F (RRID:CVCL_RW53) cells were maintained in RPMI-1640 10% FBS with the addition of 10 µM Enzalutamide for the MR49F cell line. For experimental conditions requiring hormone restriction, LNCaP/AR, LNCaP, and MR49F cells were cultured in RPMI-1640 with 10% CSS FBS. Media with phenol red was used due to weak acidity which aids in solubility of the CBs. All media contained 1xPenStrep, and 0.2% normocin (InvivoGen). For hormone restriction experiments, LNCaP/AR and LNCaP cells were hormone restricted for 24h prior to treatments, and MR49F cells were cultured in hormone restricted conditions for 72h prior to treatments. All cell lines tested *mycoplasma* negative using the MycoStrip Kit (invivogen) throughout experimental use.

RNA isolation and RT-qPCR. LNCaP/AR, LNCaP, and MR49F cells were seeded into 6-well plates and maintained in hormone restricted conditions. Cells were treated for 24h prior to collection consistent with previous approaches (Pollock et al., 2016). Total RNA was isolated using the Maxwell 16 RNA purification system (Promega; RRID:SCR_025867). cDNA synthesis was completed using the iScript Reverse transcription supermix for RT-qPCR (BioRad). AR target gene transcription was evaluated by qPCR using validated PrimePCR SYBR primers (BioRad) and SsoAdvanced Universal SYBR Green Supermix (BioRad). Data was normalized to the reference genes *TBP* and *YWHAZ*. Data represents the means ± SEM of three independent experiments performed in triplicate.

### 2.3 Immunocytochemistry and confocal microscopy

Cells were plated into sterilized coverslips coated with poly-D-lysine in six well plates and incubated in hormone deprived conditions. Cells were treated for 4h then fixed and permeabilized with 100% MeOH, blocked in 1% normal horse serum, and stained with an Anti-Androgen Receptor antibody (1:200, abcam227678, RRID:2833098). Secondary antibody incubation was completed with Donkey anti-rabbit Alexafluor 594 (1:200, Invitrogen, A-21207) and cells were counterstained with DAPI (Sigma) for nuclear DNA. Images were captured with a Nikon A1R confocal microscope (RRID:SCR_020317) through a 10 µm Z-stack (1.2 µm step) using NIS-Elements AR software. Image acquisition settings were kept consistent across treatment groups within cell lines. For nuclear localization studies, three images taken at 60x were evaluated in ImageJ for each biological replicate. The JACoP ImageJ Plug-In (RRID:SCR_003070) was used to generate the Mander’s 2 colocalization coefficients using unbiased thresholding through blinded analysis. The nuclear/cytoplasmic fraction was calculated from the proportions of nuclear and cytoplasmic AR. Data represents the means ± SEM of three independent experiments performed in triplicate.

### 2.4 MTT assay

MTT assays were performed using the Cell proliferation Kit I (Roche). Prostate cancer cells were seeded into wells at 8,000 cells/well (LNCaP and MR49F) or 2000 cells/well (LNCaP/AR). CB compounds were prepared in DMSO and applied to cells 24h after plating. The dosing range for all treatments was 100 µM–0.01 µM. MTT reagents were added to wells after 96h of drug treatment. Absorbance was measured using the Varioskan LUX Multi-Mode Microplate Reader (RRID:SCR_26792) at 562 nm and corrected by subtracting the absorbance at 650 nm. Due to precipitation of CBs at 100 µM after addition to media, data points at this concentration were excluded from analysis for all cell lines. Data represents the means ± SEM of three independent experiments performed in triplicate.

### 2.5 Molecular docking

Molecular docking studies were conducted using the Molecular Operating Environment (MOE) software from the Chemical Computing group, version MOE 2024.06.01 The crystal structure of the wild-type androgen receptor ligand binding domain in complex with S-1 (PDB 2AXA; X-ray diffraction; 1.80 Å), the T877A mutant with S-1 (PDB 2AX7; X-ray diffraction; 1.90 Å), and the F877L/T878A mutant with dihydrotestosterone (DHT) (PDB 8FH1; X-ray diffraction; 1.69 Å) were obtained from the Protein Data Bank (PDB). Prior to docking, all loaded 3D coordinates were corrected. First, polar hydrogens and partial charges were added using the “ Protonate 3D ” function of MOE. For the protonation process, a temperature of 300 K, a concentration of 0.1 mol/L salt in the solvent, and a pH of seven were specified. Secondly, any missing heavy atoms, alternate geometries, or other crystallographic artifacts were corrected using the “ QuickPrep ” function.

The ligands ClCB and BrCB were modelized from ChemDraw-generated Simplified Molecular Input Line Entry System (SMILES) and modified using the “ Builder ” tool to define fixed stereochemistry. Both ligands were protonated and energy-minimized using the AMBER10:Extended Hückel Theory (EHT) force field. These structures were included in the input docking database and subsequently docked into previously prepared AR LBD.

The flexible receptor docking was performed by specifying “ Induced Fit ” as the refinement method, which—unlike rigid docking protocols—allows localized conformational changes in the receptor active site during the generation of refinement conformations, thereby capturing the immediate structural accommodation of ligands. This approach represents an appropriate compromise between rigid docking and full molecular dynamics simulations and is well suited to the present objective of evaluating whether a conformationally constrained tetra-aryl cyclobutane scaffold can be accommodated across distinct AR mutant LBD. The number of placement poses was set to 50, and the number of generated conformations from the refinement stage was set to 5. The charges of all atoms were reassigned under the EHT force field. Final pose scoring was performed based on the London dG free binding energy. For further analysis of the ligand-receptor interactions, the docked poses with the lowest London dG values were chosen.

“ Align Sequences and Superpose ” function of MOE allowed representation of the androgen receptor ligand-binding domains from the same angle. “ Measure distances ” function of MOE was used to calculate the interatomic distance between two selected atoms, with the corresponding atoms specified in the manuscript and all distances reported in angstroms (Å).

### 2.6 Statistics

Ordinary one-way ANOVAs and two-way ANOVAs with Dunnett’s multiple comparisons test, and nonlinear regression were performed using GraphPad/Prism version 10.5.0 (GraphPad Software Inc., RRID:SCR_002798). Graphs in Figures 2–4 (2 B–D, 3G, 4B–C) represent sample mean ± SEM n = 3. Significance is represented by *p ≤ 0.05, **p ≤ 0.01, ***p ≤ 0.001, and ****p ≤ 0.0001. Nonlin Fit was used to determine IC50 values in Figure 4A.

**FIGURE 1:**
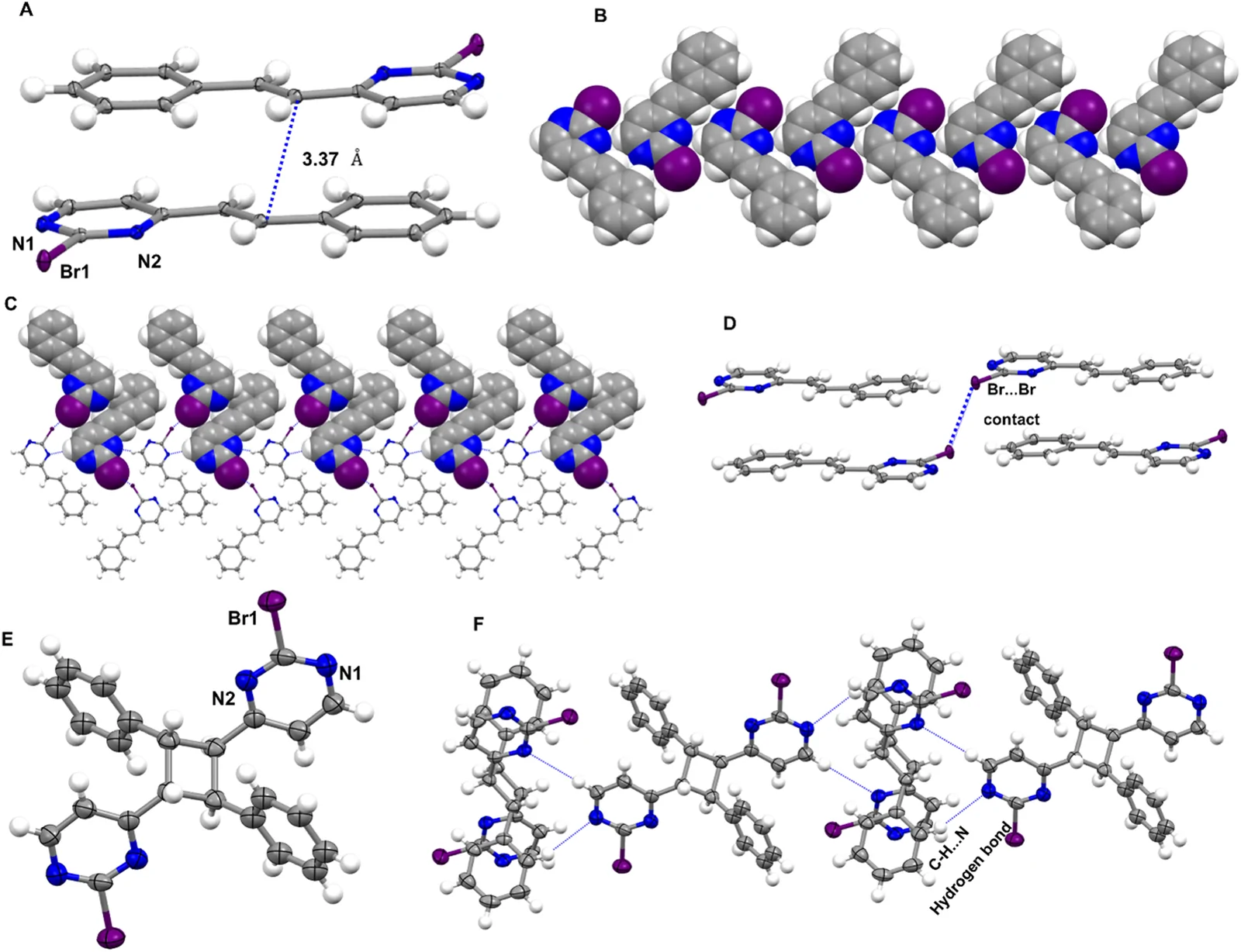
Crystal structures BrSP and BrCB **(A)** Head-to-tail dimer showing the stacking of the C=C bonds of the alkene with a 3.37 Å distance **(B)** 1D edge-to-edge view of sheets encompassing neighboring molecules of BrSP supported by C-H‧‧‧N hydrogen bonds **(C)** 2D C-H‧‧‧N and Br‧‧‧Br intermolecular interactions **(D)** Br‧‧‧Br halogen bond of the alkene of the nearest neighbor BrSP **(E)** ring of BrCB highlighting the rctt stereochemistry, and **(F)** extended packing of the BrCB showing the interaction of the nearest neighbor through two C-H‧‧‧N hydrogen bonds. Corresponding color identifiers for atoms include hydrogen (white), carbon (grey), nitrogen (blue) and bromine (purple). All intermolecular interactions are indicated by dotted blue lines.

**FIGURE 2:**
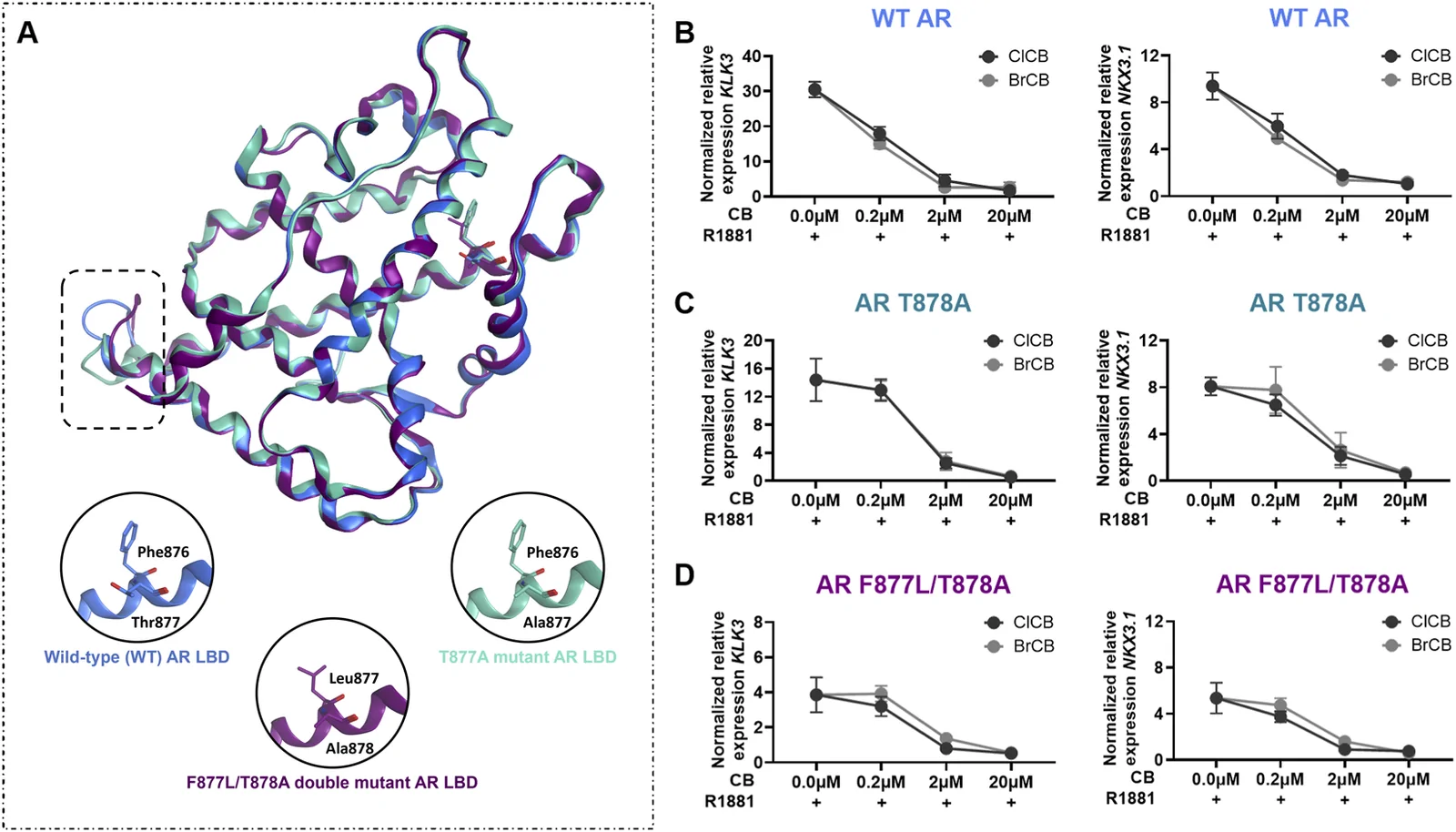
Dose dependent transcriptional inhibition by CBs does not translate to impacts on cell viability **(A)** Structural overlay of the WT (blue), T877A mutant (cyan), and F876L/T877A double mutant (purple) AR LBDs. Disordered overlapping regions are highlighted in a black square. The three LBDs are representative of the structural changes to AR in the LNCaP/AR, LNCaP, and MR49F cell lines **(B–D)** qPCR quantification of *KLK3* or *NKX3.1* transcript expression after 24 h treatment with 0.2 μM, 2 μM, and 20 μM ClCB or BrCB in the presence of 1 nM R1881. Observations are reported for LNCaP/AR (ARWT), LNCaP (ART878A) cells, and MR49F (ARF877L/T878A) cell lines respectively. Dose dependent inhibition is observed for both CBs in each of the AR LBD point mutants.

**FIGURE 3:**
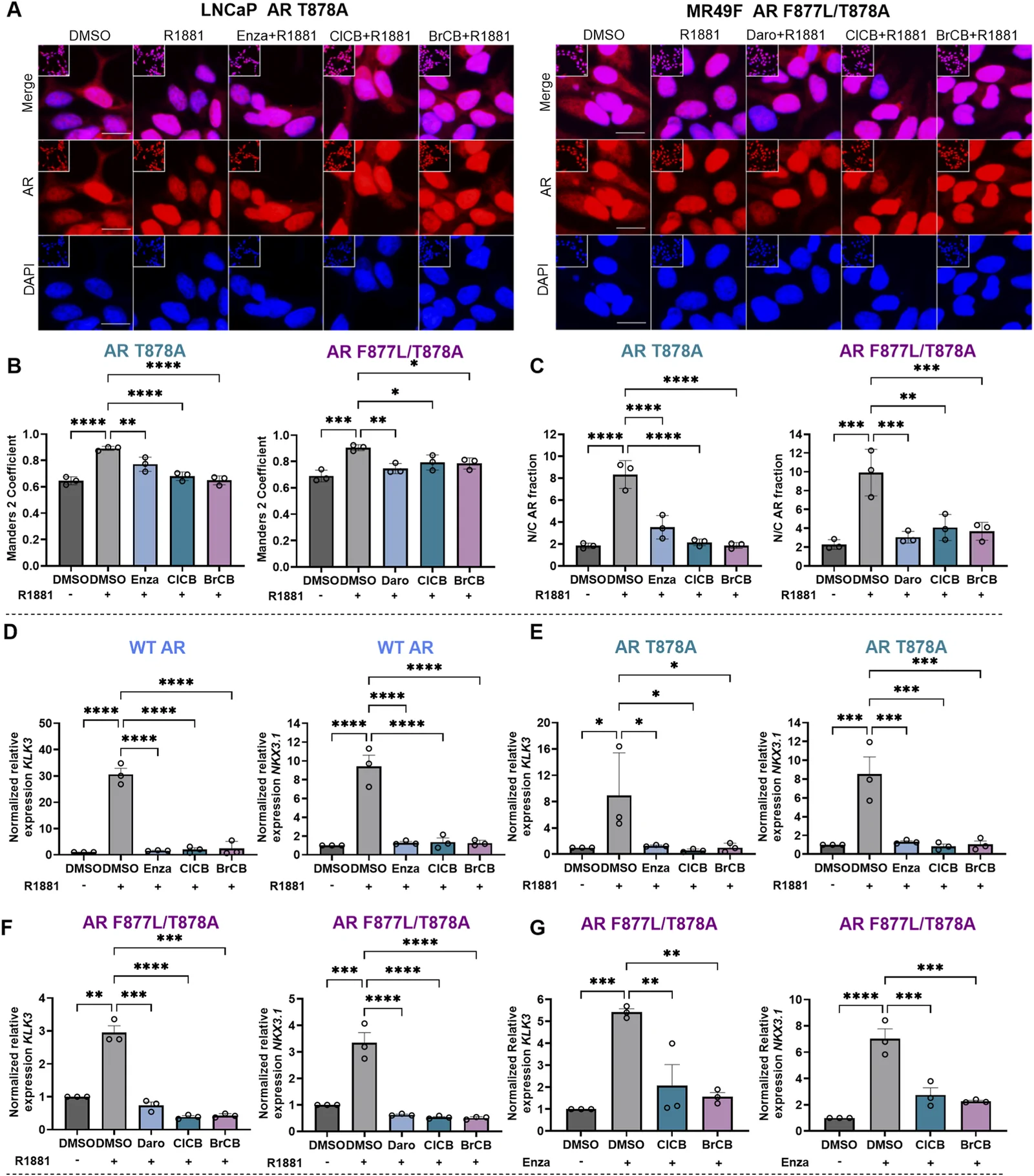
BrCB mechanism of action is consistent with ClCB and analogous to the impacts of traditional ARPIs **(A)** Representative confocal ICC images of LNCaP (ART878A) cells and MR49F (ARF877L/T878A) after 4h treatment with 20 μM CBs or ARPIs. AR protein (red) and DAPI as a counterstain for nuclear area (blue). Scale bar = 20 μm **(B)** Quantification of AR protein colocalized with DAPI represented with the Mander’s 2 coefficient as the proportion of nuclear AR in LNCaP and MR49F cells. Treatment with CBs blocks nuclear internalization of AR induced by R1881 **(C)** Secondary validation of cellular AR protein localization assessed using the nuclear/cytoplasmic fraction (N/C fraction) of AR staining **(D–F)** *KLK3* and *NKX3.1* gene expression demonstrating blockade of the AR signaling pathway after 24 h combination treatment with 20 μM ClCB or 20 μM BrCB +1 nM R1881 compared to the FDA approved ARPI 20 μM + 1 nM R1881 in the LNCaP/AR (ARWT), LNCaP (ART878A) cells, and MR49F (ARF877L/T878A) cell lines **(G)** Quantification of gene expression downstream of AR in MR49F ARF877L/T878A cells where transcription was induced with 20 μM Enza and subsequently blocked by cotreatment of 20 μM CBs + 20 μM Enza. One way ANOVA with Dunnett’s multiple comparisons test error bars represent mean ± SEM (n = 3). Significance is represented by *p ≤ 0.05, **p ≤ 0.01, ***p ≤ 0.001, and ****p ≤ 0.0001.

**FIGURE 4:**
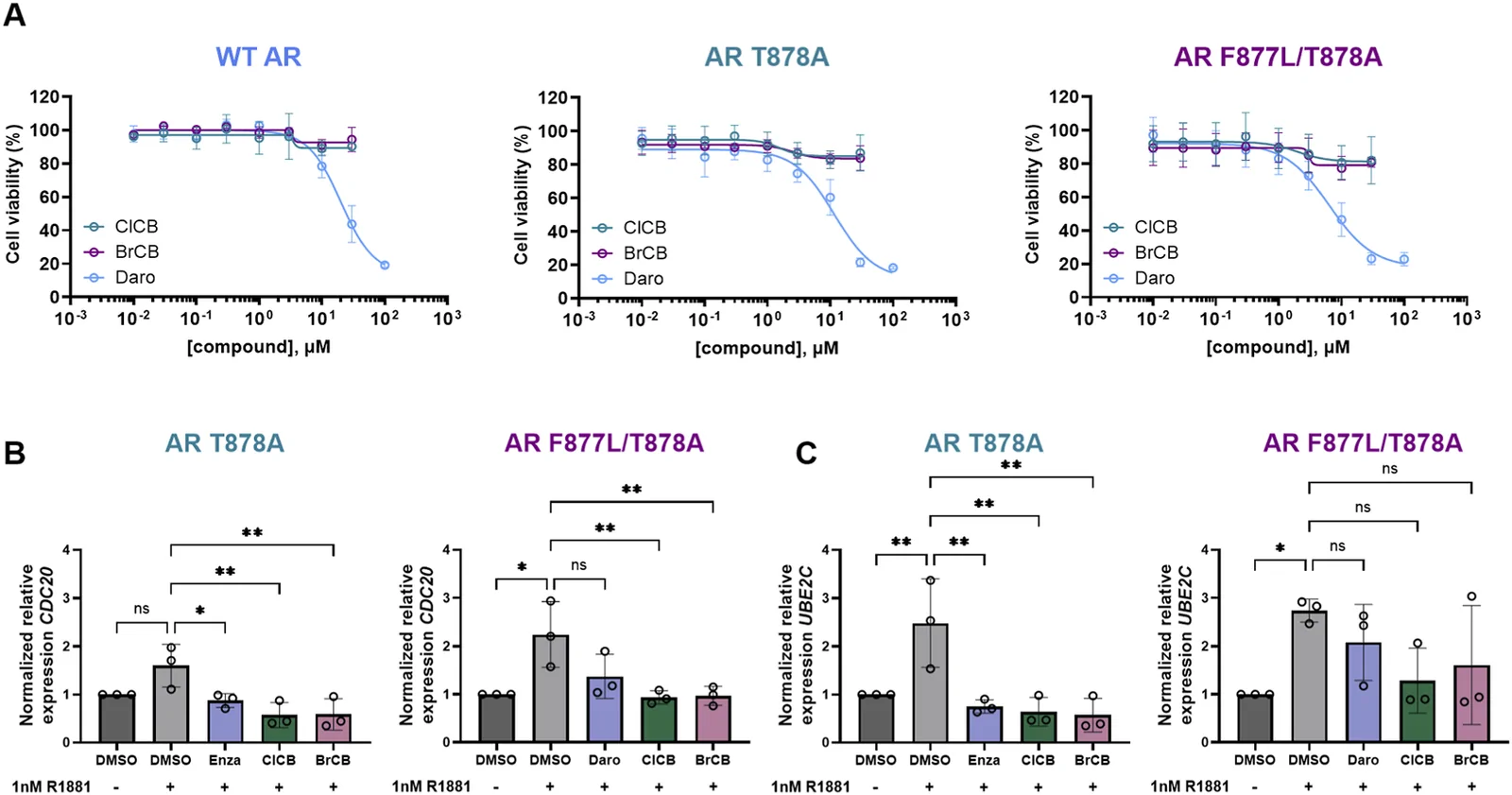
CBs exhibit characteristics of cytostatic agents **(A)** MTT assay data evaluating percent cell viability in all cell lines after 96h of exposure to increasing concentrations of CBs or Daro. A dose response curve is observed with Daro treatment but not with CB treatment **(B)** Quantification of *CDC20* gene expression in the LNCaP and MR49F demonstrating a reduction in cell division compared to R1881 after 24 h treatment **(C)** Expression of the cell cycle regulator *UBE2C* decreased compared to R1881 after 24 h treatment in the LNCaP but not the MR49F cell line. MTT assay was evaluated via nonlinear regression. IC50 values were not detected for BrCB and ClCB treatments (n = 3). One way ANOVA with Dunnett’s multiple comparisons test error bars represent mean ± SEM (n = 3). Significance is represented by *p ≤ 0.05, **p ≤ 0.01, ***p ≤ 0.001, and ****p ≤ 0.0001.

**SCHEME 1:**
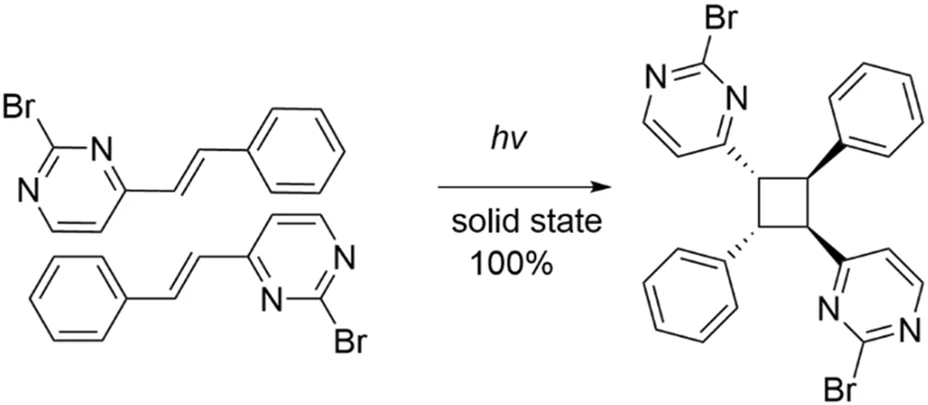
2 + 2 photodimerization of BrSP to generate BrCB in the solid state.

## 3 Results

### 3.1 Synthesis and crystal engineering of CBs

To expand our exploration of tetra-aryl cyclobutanes as candidate AR inhibitors, we synthesized the -Br styrylpyrimidine (BrSP) (Scheme1; Supplementary Figures S1-S4). For an alkene to react in the solid state to form a CB, the carbon-carbon double (C=C) bonds are expected to be organized within 4.2 Å and aligned parallel (Figure 1A; Supplementary Figure 16A). We made use of the idea of steric replacement in crystal engineering to pursue the synthesis of BrSP, which would react in the solid state to form BrCB (Supplementary Table S1). Crystalline BrSP was determined to be photoreactive to generate the corresponding head-to-tail *rctt* isomer BrCB (Supplementary Figure 1E; Supplementary Figures S5-S7) as the solid product (Scheme 1). The crystal structures of Cl- and BrSP are isostructural. The work also describes the characterization and X-ray crystal structure of the previously synthesized ClCB (Supplementary Figure 14; Supplementary Figures S10-S11-S15, S16E). In the solid state, BrSP forms one-dimensional (1D) assemblies held together by C-H … N hydrogen bonds involving the pyrimidine moieties (Supplementary Figures 1B,C,16B,16C). X … X halogen bonds effectively align the 1D assemblies such that the C=C bonds are separated by 3.37Å and fall within the geometry criteria for a [2 + 2] photodimerization (4.2Å and parallel) (Supplementary Figure 1D: Supplementary Figure 16D). UV-irradiation of BrSP results in quantitative conversion of the solid to BrCB (Supplementary Figure S5). (Supplementary Figure S7). The CB in the solid state was determined to interact with its neighbor through C-H … N hydrogen bonds to form planar sheet (Supplementary Figure 1F; Supplementary Figure 16F). We emphasize the utility of generating and profiling the BrCB as the compound also offers distinct potential both as a standalone drug candidate and as a base scaffold for future chemical optimization (e.g., Suzuki coupling) (Schomaker and Delia, 2001). The crystallinities of the alkene intermediates and the CBs were confirmed by PXRD, where agreement between the calculated and experimental diffractograms showed that the single crystals were representative of the bulk materials (Supplementary Figures S12–S15). HRMS (ESI^+^) analyses supported the structures (Supplementary Figure S4; Supplementary Figure S7).

### 3.2 The ClCB and derivative BrCB exhibit potent dose dependent transcriptional inhibition

Previous ClCB studies were conducted using AR transfection models and a mixture of PCa cell lines (Pollock et al., 2016). However, much of this work utilized models where point mutated ARs were not expressed endogenously. Our goal is to prioritize consistent model systems where AR and downstream cellular machinery will be most representative of the CRPC cellular environment. To examine each ligand-receptor complex *in vitro*, we employed CRPC cell lines that endogenously express AR point mutants. The LNCaP (AR^T878A^) was chosen to represent single point mutation (Abate-Shen and Nunes De Almeida, 2022; Horoszewicz et al., 1980). The MR49F (AR^F877L/T878A^) cell line—a derivative of LNCaP—was used to represent broader drug resistance to two ARPIs (Coleman et al., 2016). This is the first report for the application of CBs to CRPC models with co-occurrent AR LBD point mutation. For AR^WT^, an LNCaP derivative cell line (LNCaP/AR) was used where a codon switch was induced to express AR^WT^ (Tran et al., 2009). Functional validation confirmed that LNCaP/AR cells were susceptible to inhibition by flutamide due to loss of T878A point mutation, and enzalutamide was demonstrated to induce transcriptional activity in the MR49F cell model where AR^F877L/T878A^ is present (Supplementary Figure S17).

Structural comparison of the AR LBDs present in our cell lines of interest showed that the LBD of the wild-type (WT) AR, located at its C-terminal region, comprises 11 α-helices, 4 β-sheets, and 14 turns. The T878A mutant exhibits a structural rearrangement resulting in the presence of 12 α-helices and 15 turns, while the F877L/T878A double mutant displays 16 turns. Structural superposition of the WT (blue), T877A (cyan), and F877L/ T878A (purple) AR LBDs reveals conformational deviations localized within the disordered loop region (Figure 2A). The structural deviations highlight the conformational flexibility of the AR LBD induced by point mutations, which may influence ligand binding and receptor activation.

Hallmarks of AR activation include transcription of *KLK3* and *NKX3.1* target genes. Quantification of gene expression determined the dose of ClCB and BrCB necessary for complete inhibition of AR regulated transcription in each cell line. In all three cell lines, dose dependent inhibition of AR target gene expression was observed for the ClCB and BrCB (Figures 2B–D).

### 3.3 CBs function to inhibit AR nuclear internalization and transcriptional activity

The LNCaP (AR^T878A^) and MR49F (AR^F877L/T878A^) models were chosen to evaluate CBs influence on cellular processes downstream of therapeutic resistant AR. Importantly, both the LNCaP and MR49F cellular models endogenously express their AR point mutants and exhibit AR amplification which often co-occurs clinically along with point mutation (Ledet et al., 2020). These models will provide the most representative *in vitro* systems for evaluating downstream molecular mechanisms.

One of the primary methods anti-androgens inhibit AR is through a blockade of nuclear internalization (Tran et al., 2009; Chen et al., 2009). As reported for the ClCB (Pollock et al., 2016), we hypothesized that blockade of AR nuclear translocation would also occur with the BrCB owing to the structural similarity of the two compounds. LNCaP and MR49F cells were stained via fluorescence immunocytochemistry for AR and counterstained for nuclei with DAPI. Confocal imaging and analysis of LNCaP and MR49F cells showed that AR was ∼35% cytoplasmic and ∼65% nuclear at baseline in both models. Treatment with R1881 stimulated nuclear internalization of ARs, and cotreatment of R1881+CB or ARPI halted this effect in both cell lines. (Figure 3A). Quantification of cell compartmentalization by colocalization of ARs to DAPI was measured with the Mander’s 2 coefficient (Figure 3B) and the nuclear/cytoplasmic fraction of ARs (Figure 3C). Together, these findings indicate that CBs block AR nuclear internalization and that progression from isolated to co-occurrent point mutation does not hinder the CB mechanism of action.

Independent studies on each cell line were completed to gauge the inherent activity of each ligand as a full or partial agonist or antagonist. Representative images and quantification of AR localization in each cell line was indicative of complete antagonistic properties for both the ClCB and BrCB (Supplementary Figures S18, S19). Consistent with expectations, R1881 and Flut promoted nuclear internalization of AR in LNCaP cells while R1881, Flut, and Enza increased nuclear AR in MR49F cells. All other ligands did not induce substantial changes in AR cell compartmentalization. (Supplementary Figures S18,S19).

Our work compared CBs at matched concentrations of 20 μM to ARPIs. Enza was used as the control ARPI in LNCaP and LNCaP/AR cell lines, and Daro was used as the control ARPI for MR49F cells. Consistent with expectations, treatment with R1881 induced the expression of *KLK3* and *NKX3.1* and co-treatment of R1881+CBs blocked transcript expression with similar efficacy to traditional ARPIs (Figures 3D-F). Upon exposing the Enza resistant MR49F cell line to 20 μM Enza, AR signaling was activated, and CBs were demonstrated to outcompete Enza and block transcription downstream of AR (Figure 3G). These findings highlight CBs as drug candidates with comparable inhibitory efficacy to second generation ARPIs.

### 3.4 CBs exhibit characteristics of cytostatic rather than cytotoxic agents

A characteristic impact of AR inhibition in AR dependent cells is loss of cell viability. We expected that dose dependent inhibition of AR target gene transcription would render a similar trend in cell viability when measured by MTT assay. LNCaP/AR, LNCaP, and MR49F cells were treated with 0.01–100 µM ClCB, BrCB, or Daro to evaluate cell viability after 96 h of treatment (Figure 4A). Surprisingly, cells treated with CBs retained their viability up to the maximum solubility of CBs at 30 µM in media. Matched concentrations of Daro produced the expected dose response curve and reduced viability with increasing concentration in all three cell lines. These data demonstrate that CBs exert distinct impacts on cell viability when compared to traditional ARPIs despite potent AR transcriptional inhibition. This finding points to potential cytostatic rather than cytotoxic activity of CBs. To further evaluate this hypothesis, we examined the cell cycle markers *CDC20* and *UBE2C* since cytostatic agents prevent cancer progression through inhibiting cell growth and division rather than inducing cell death. Quantification of *CDC20* gene expression in the LNCaP and MR49F cell lines after CB treatment demonstrated a reduction in the mRNA encoding *CDC20*, an essential regulator of cell division (anaphase initiation), compared to active cycling induced by R1881 (Figure 4B). The cell cycle regulator *UBE2C* was also demonstrated to decrease compared to R1881 after CB treatment in the LNCaP cell line, but not the MR49F cell line (Figure 4C). Together, this data supports the cytostatic functionality of CBs where cell growth is reduced but cell viability is retained compared to cytotoxic agents like ARPIs.

### 3.5 Molecular docking and comparison of CB binding poses within AR LBD point mutants

As CBs exhibit a distinct structure and cellular impact from both first- and second-generation ARPIs, we sought to define CB/AR ligand-receptor complexes. As reported, resistance-associated point mutations within the AR LBD—notably T878A and F877L—can confer therapeutic resistance by altering ligand recognition or modulating receptor activation. In some cases, resulting in a functional switch from antagonist to agonist behavior (Liu et al., 2016; Liu et al., 2017a; Liu et al., 2017b). In the present study, molecular docking was carried out to elucidate the binding modes of ClCB and BrCB ligands within the wild-type (WT) AR and two mutants, and to systematically evaluate the impact of resistance mutations on binding of both ligands. Literature on AR point mutations can differ in annotation based on codon counts used. Therefore, Phe can be located at either the 876 or 877 codon and Ala can occur at either the 877 or 878 codons. F876 L and F877 L are equivalent point mutations, as are T877A and T878A. Our molecular docking figures use codon counts consistent with the PDB files used for analysis. The selection of receptor structures was based on previously reported AR LBD crystal structures, further compiled in comparative reports (Gim et al., 2021; Colah et al., 2026) prioritizing high-resolution entries with bound small-molecule ligands and the availability of experimentally resolved clinically relevant AR mutants. Accordingly, we employed the crystal structure of the WT AR LBD in complex with S-1 (PDB 2AXA), the T877 A mutant with S-1 (PDB 2AX7), and the F877L/T878 A mutant with dihydrotestosterone (DHT; PDB 8FH1). The previously validated London dG scoring function (PMID: 40649292) was applied to prioritize energetically favorable ligand-binding poses for comparative analysis.

Hydrophobic contacts, aromatic interactions and hydrogen bonding (H-bond) networks established between the ligands (ClCB and BrCB) and ARs were analyzed in depth using the “Protein Contacts” function in MOE software to give, with three-dimensional (3D) representations (Figure 5) and complementary two-dimensional (2D) ligand interaction diagrams (Supplementary Figure S20). Both ligands were found to occupy the lipophilic pocket of ARs, predominantly formed by nonpolar amino acid residues (i.e., Leu, Phe, Trp and Val) (Figure 5; Supplementary Figure S20). This spatial arrangement suggests that weak, non-directional Van der Waals (VdW) forces constitute the principal energetic contributors to ligand–receptor complex formation. Given the pronounced ligand hydrophobicity, the binding process is primarily entropically driven, thereby conferring a thermodynamically favorable interaction profile. Nonetheless, in some cases, additional stabilizing, directional interactions—such as H-bonds and aromatic interactions—were also determined upon docking (Figure 5; Supplementary Figure S20). For example, in the AR^WT^, the Cl-pyrimidyl moiety of ClCB participates in weak H-bonding interactions with Met742 (d_H-SD_ = 3.00 Å) and Met787 (d_H-SD_ = 2.64 Å), each contributing to an estimated interaction energy of −0.50 kcal/mol (Figure 5A; Figure 6A; Supplementary Figure S20A). Structural superposition of the ClCB ligand in both the WT and T877A mutant AR LBDs reveals a highly conserved binding mode, characterized by the co-localization of the phenyl rings and the electron-deficient heteroaryl moieties within the binding pocket (Figure 6A; Figure 6C). In contrast, within the F877L/T878A double mutant AR, the Cl-pyrimidyl group engages exclusively in a weak hydrogen bond with Met788 (−0.80 kcal/mol, d_H-SD_ = 2.46 Å), failing to establish contact with Met743 (Figure 6B; S20E; Tables S2a-d).

**FIGURE 5:**
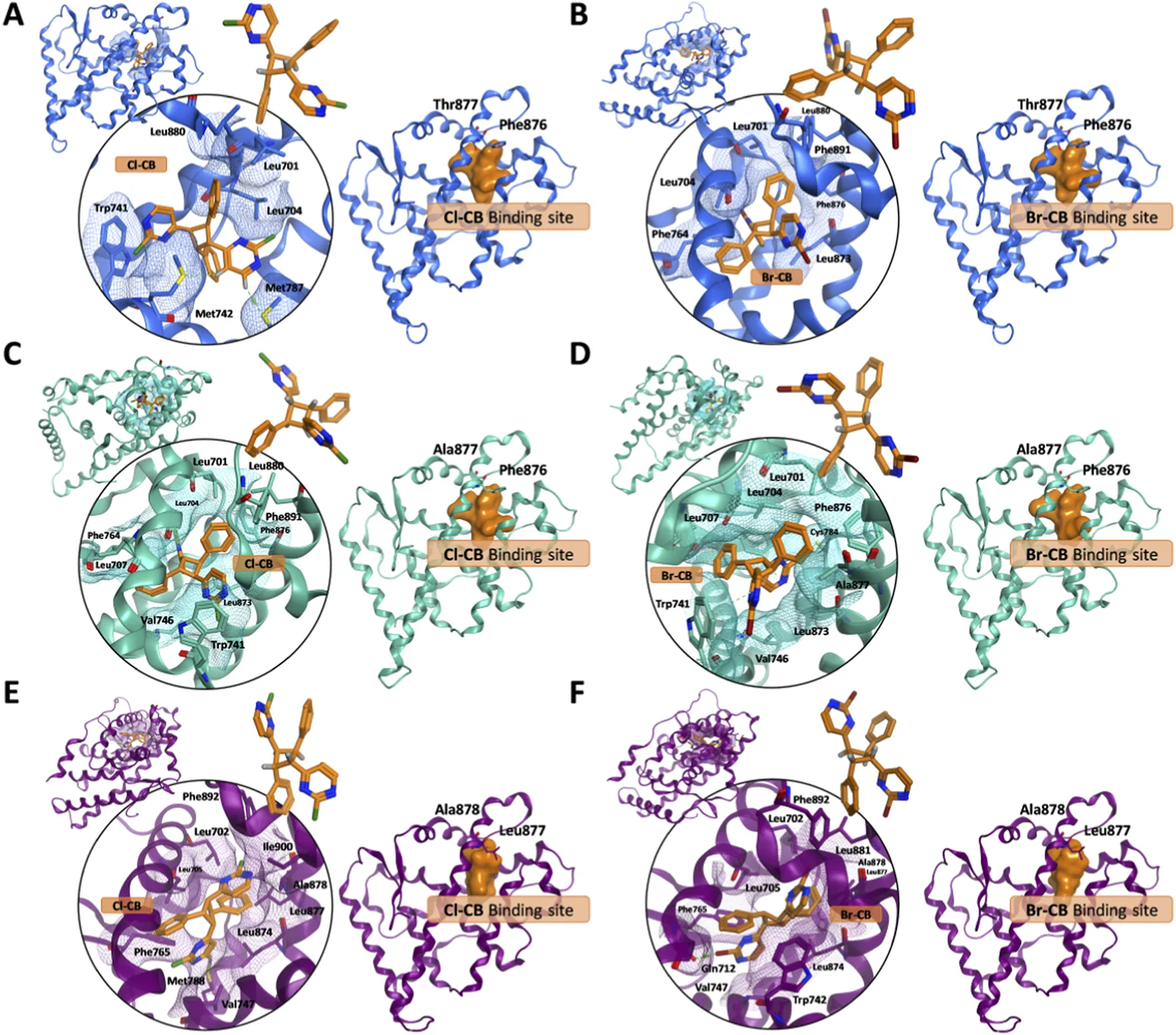
Docking poses of CBs are energetically favorable in LBDs of AR WT, ART877A, and ARF877L/T878A. Comparative analysis of ClCB and BrCB docking poses in the **(A,B)** WT (blue) **(C,D)** T877A mutant (cyan), and **(E,F)** F877L/T878A double mutant (purple) AR LBDs. Black circles highlight the detailed binding interactions of ClCB and BrCB within the lipophilic pocket of the AR LBDs. Sidechains of residues involved in hydrophobic interactions with the ligands are represented as bold sticks in blue, cyan, or purple, corresponding to each receptor variant, and are annotated accordingly. The spatial contour of the lipophilic pocket accommodating each ligand is visualized as a semi-transparent mesh. H-bonds are illustrated as green dashed lines with a strength bar, while aromatic interactions are shown as light green dashed lines. Each annotated docking pose is paired with a surface model depiction for each respective ligand providing spatial context for their interactions within the ribbon representation of the receptor. F876L/F877L and T877A/T878A are highlighted as bold sticks in the AR mutants and the corresponding WT receptor, emphasizing their structural positioning within the binding domain. Note: F876L/F877L and T877A/T878A are used interchangeably to annotate point mutations throughout the literature dependent on method of codon count.

**FIGURE 6:**
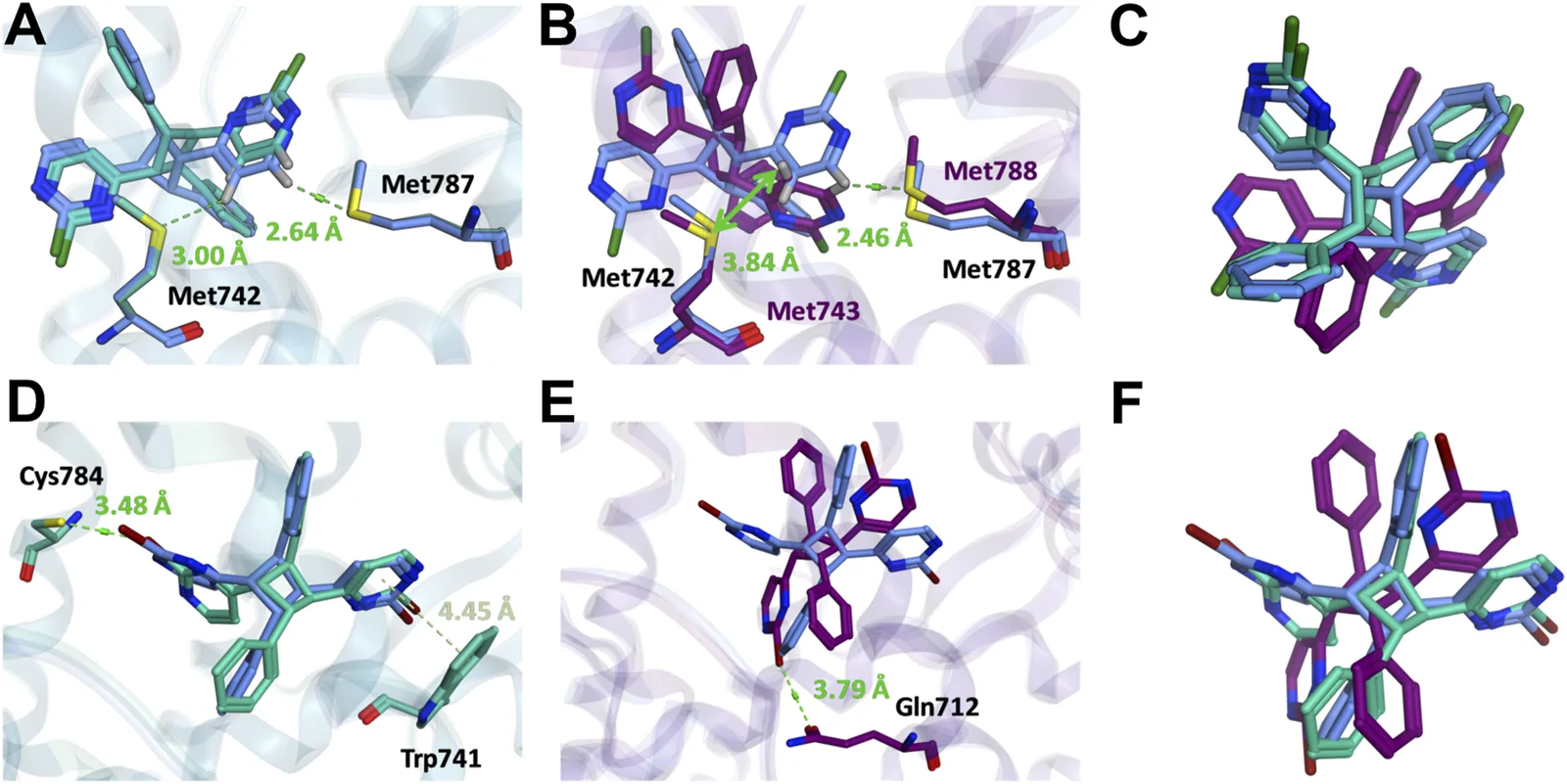
Comparative binding poses of ClCB and BrCB within AR LBD point mutants **(A,D)** Structural superposition of ClCB and BrCB docking poses within the wild-type (WT, blue) and T877A mutant (cyan) AR LBDs. The off-set stacking aromatic interaction is indicated by a light green dashed line **(B,E)** Superposition of ClCB and BrCB ligand docking conformations within the WT (blue) and F877L/T878A double mutant (purple) AR LBDs. The spatial distance from the hydrogen atom of the Cl-pyrimidyl moiety in the docked ClCB to Met743 is indicated by a green bidirectional arrow. Predicted H-bonding and VdW interactions between ligands and AR LBD variants are illustrated as green dashed lines with a strength bar, with the associated bond distances labeled in Å. The AR LBDs are rendered in transparent ribbon representations to emphasize ligand orientations across the AR LBD variants **(C,F)** Overlay of docked ClCB and BrCB conformations across all three AR LBD variants: WT (blue), T877A mutant (cyan), and F877L/T878A double mutant (purple).

The docking poses of ClCB and BrCB in both the WT and T877A single mutant AR LBDs revealed a high degree of structural similarity, indicating that the Thr877 to Ala877 substitution exerts minimal influence on ligand orientation or binding site occupancy (Figures 6A,B,D,E). In contrast, docking within the F877L/T878A double mutant AR LBD demonstrated a notable conformational shift in both ligands. This is attributable to altered steric constraints introduced by the combined mutations F877L/T878A, which reshape the binding cavity and induce ligand reorientation (Figures 6C,F). The “ Induced Fit ” docking calculations offer foundational insight into the predicted local interactions between CBs and the AR LBD across distinct point mutants. Based on these binding modes, informed design can guide the synthesis of BrCB and other halogenated derivatives with enhanced pharmaceutical potential. Strategic functionalization should continue to prioritize ligands that maintain antagonism even across AR isoforms. This unique property of CBs is an unconventional advantage that fosters a new perspective on rational drug design for CRPC.

## 4 Discussion

Here, we describe the application of CBs in models of CRPC, involving both isolated and co-occurrent AR point mutation, to examine the ability of CBs to bypass mechanisms of therapeutic resistance. Our results led us to consider CBs as cytostatic rather than cytotoxic agents. Consistent with the expectations of cytostasis, CBs treatment induced markers of slowed growth in CRPC cells without inducing apoptosis at concentrations sufficient to block AR activity. Moreover, CBs accommodate structural changes in the AR LBD through altered binding orientation as demonstrated when modeled in AR progression from isolated to co-occurrent LBD point mutation. This molecular feature is highly desirable and could offer the first step in optimization of an adaptive AR inhibitor that is less susceptible to the pharmacological impacts of point mutation. However, additional structural biology experimentation is necessary to further validate CBs as adaptive ligands. Collectively, our findings demonstrate that strategic derivatization of CBs, using crystal engineering principles, can expand this distinct class of AR inhibitors. CB scaffolds harbor unique pharmaceutical potential which should continue to be explored using rational drug design tactics. Our work endorses CBs as desirable therapeutic candidates in CRPC and other androgen driven diseases and disorders.

Crystal engineering is emerging as a technique to synthesize functional materials with applications in pharmaceutics, energy storage, agriculture, and electronics (Nangia and Desiraju, 2019). Reactions in crystals are now providing access to molecules difficult to obtain using conventional (solution-based) organic synthesis. Solid state reactions help to perform organic syntheses that are efficient, and atom-economical (Freund et al., 2018). Given that CB products are generated quantitatively, without side products, and with pharmacological activity against AR, it is evident that this is a vital development in synthesizing therapeutic agents for CRPC. Since capitalizing on thoughtful, resource efficient, and environmentally conscious synthesis is becoming a priority in academic research and industries (Freund et al., 2018), research that emphasizes solvent-reduced and solvent-free reactions is important (Freund et al., 2018). Considering CBs as potential candidates in the pharmaceutical development pipeline, the [2 + 2] photodimerization reaction (Scheme 1), inherently incorporates solvent-free synthesis. Notably, this reaction is accomplished through UV radiation, is atom economical, and produces a nearly pure product with no byproducts.

Thus far, application of crystal engineering to the pharmaceutical industry has generated a class of multi-component drugs known as cocrystals (Bolla et al., 2022). Accumulating successes of cocrystals highlights potential for crystal engineering to impact current approaches to rational drug design (Bolla et al., 2022). However, crystal engineering of reactions in the solid state that allow for the development of single component drug molecules has yet to gain similar traction. To date, publications leveraging crystal engineering techniques in pharmaceutical development for CRPC are limited. Therefore, it is significant to report that the therapeutic candidates ClCB and the newly derived BrCB are generated using crystal engineering. Moreover, abundant opportunity remains to further optimize CBs through the combination of structure activity relationship analysis and crystal engineering.

In the context of CRPC, CBs are attractive drug candidates with thermodynamically favorable interaction profiles in AR LBDs. Previously reported X-ray crystal data have allowed for close examination of the structure activity relationships between each CB paired with AR^WT^, AR^T878A^, and AR^F877L/T878A^. Both the ClCB and BrCB take on a distinct ligand binding orientation in context of co-occurrent point mutation (Figure 5). Historically, molecular docking studies have shown that ligand reorientation of ARPIs including flutamide, bicalutamide, and enzalutamide leads to a loss of function in respective point mutated AR LBDs (Liu et al., 2015; Liu et al., 2016; Liu et al., 2017a; Liu et al., 2017b). The functional shift is primarily a result of the inability of ARPIs to sterically hinder helix 12 (H12) from closing after point mutation. Once closed, H12 forms a coactivator binding site necessary for transcription (Liu et al., 2015; Liu et al., 2016; Liu et al., 2017a; Liu et al., 2017b). Despite co-occurrent point mutation inducing reorientation of the CBs, we find that complete inhibition of AR transcriptional activity (AR target genes *KLK3* and *NKX3.1*) and blockade of AR nuclear internalization still occurs (Figures 2, 3). This remarkable finding indicates that the structure of CBs offers an adaptive inhibitory profile in contexts where traditional ARPIs are prone to failure. Although our work has yet to profile H12 dynamics in the context of CB/AR complexes, we propose this area as a next critical component necessary to optimize CB scaffolds as therapeutic candidates in CRPC. Moreover, additional cryo-electron microscopy (cryo-EM) studies will be required to further support the proposed adaptive ligand-binding mode of CBs.

During our profiling of the CBs in models of AR LBD point mutants, we predicted–based on the foundational biological concept that form dictates function–that BrCB would exhibit major functional overlaps with the ClCB. Comparable pharmacological profiles were observed for the two compounds. Overlapping capacities include pan-inhibition of AR LBD point mutants despite ligand reorientation, dose-dependent AR inhibition, blockade of AR transcriptional activity and AR nuclear entry, and minimal impacts on cell viability. The absence of an impact on cell viability led us to consider cytostatic rather than cytotoxic functions of CBs. Consistent with expectations of cytostatic agents, the cell markers *CDC20* and *UBE2C* were reduced after CB treatment demonstrating slowed growth. This finding was consistent with previous reports demonstrating that the ClCB suppresses cellular proliferation *in vitro* (Pollock et al., 2016). Cofactor profiling of AR/ClCB complexes offers a plausible explanation for retention of cell viability after ligand binding (Pollock et al., 2016). The AR/ClCB ligand complex was found to be conformationally similar to an unliganded-like receptor and did not cluster with the receptor conformation induced by traditional first- or second-generation ARPIs (Pollock et al., 2016). Therefore, cellular mechanisms downstream of AR cofactors that reduce viability after ARPI treatment may not be triggered when CBs bind to AR. This concept provides an interesting route for future investigation of how the structural overlap between the ClCB and BrCB guides similar cellular mechanisms while simultaneously remaining distinct from traditional ARPIs.

To highlight the pharmaceutical potential of CBs across the diverse mutational landscape of the AR LBD, we reiterate the originality of deciphering a structure that can effectively bind to the AR LBD in multiple orientations while retaining antagonistic function and potency. Moreover, our molecular docking studies provide a basis for ligand optimization from the CB scaffold. Computational data implies that binding affinity and selectivity could be significantly enhanced through strategic functionalization at the ortho/para-position of the phenyl ring of CBs with H-bond acceptor/donor groups. These conclusions highlight both immediate and continued potential for the solid-state development of CB derivatives with distinct pharmaceutical potential.

Our work is timely because many clinically prevalent and emerging AR LBD targeting therapeutics prioritize potency above all else (Borgmann et al., 2018; Crawford et al., 2018; Hussain et al., 2018). This technique for novel therapeutic development has resulted in the cyclical failure of ARPIs since potent selective pressure favors the emergence of resistance mechanisms (Steketee et al., 2002; Veldscholte et al., 1990; Hara et al., 2003; Korpal et al., 2013). Through our work, we hope to foster the development of AR LBD targeting therapeutics which prioritize ligand adaptability to enhance long-term efficacy. We anticipate this new approach will yield a class of ligands with distinct potential to improve patient outcomes. We also anticipate that our application of green chemistry will support a rise in sustainable approaches to synthesis within the pharmaceutical industry, making our work to prioritize the approaches in the rational design of CBs increasingly relevant.

## Data Availability

The crystal data that supports the findings of this study are openly available in Cambridge Structural Database at https://www.ccdc.cam.ac.uk, reference numbers 2484979, 2484980, 2484981, and 2484982. All other data are included in the manuscript or Supplementary Material.

## References

[B1] Abate-Shen C. Nunes De Almeida F. (2022). Establishment of the LNCaP cell line – the dawn of an era for prostate cancer research. Cancer Res. 82, 1689–1691. 10.1158/0008-5472.CAN-22-1065 35502546 PMC9153264

[B2] Beltran H. Yelensky R. Frampton G. M. Park K. Downing S. R. Macdonald T. Y. (2013). Targeted next-generation sequencing of advanced prostate cancer identifies potential therapeutic targets and disease heterogeneity. Eur. Urol. 63, 920–926. 10.1016/j.eururo.2012.08.053 22981675 PMC3615043

[B3] Bolla G. Sarma B. Nangia A. K. (2022). Crystal engineering of pharmaceutical cocrystals in the discovery and development of improved drugs. Chem. Rev. 122, 11514–11603. 10.1021/acs.chemrev.1c00987 35642550

[B4] Borgmann H. Lallous N. Ozistanbullu D. Beraldi E. Paul N. Dalal K. (2018). Moving towards precision urologic oncology: targeting Enzalutamide-resistant prostate cancer and mutated forms of the androgen receptor using the novel inhibitor darolutamide (ODM-201). Eur. Urol. 73, 4–8. 10.1016/j.eururo.2017.08.012 28851578

[B5] Carreira E. M. Fessard T. C. (2014). Four-membered ring-containing spirocycles: synthetic strategies and opportunities. Chem. Rev. 114, 8257–8322. 10.1021/cr500127b 25003801

[B6] Chen Y. Clegg N. J. Scher H. I. (2009). Anti-androgens and androgen-depleting therapies in prostate cancer: new agents for an established target. Lancet Oncol. 10, 981–991. 10.1016/S1470-2045(09)70229-3 19796750 PMC2935850

[B7] Colah A. Ferková S. Zhang H. Liu G. Macgillivray L. Boudreault P. L. (2026). Androgen receptor point mutations: a mechanism of therapeutic resistance and a framework for rational drug design. Cancers (Basel) 18, 1043. 10.3390/cancers18061043 41899643 PMC13025137

[B8] Coleman D. J. Van Hook K. King C. J. Schwartzman J. Lisac R. Urrutia J. (2016). Cellular androgen content influences enzalutamide agonism of F877L mutant androgen receptor. Oncotarget 7, 40690–40703. 10.18632/oncotarget.9816 27276681 PMC5130036

[B9] Crawford E. D. Schellhammer P. F. Mcleod D. G. Moul J. W. Higano C. S. Shore N. (2018). Androgen receptor targeted treatments of prostate cancer: 35 years of progress with antiandrogens. J. Urol. 200, 956–966. 10.1016/j.juro.2018.04.083 29730201

[B10] Culig Z. Santer F. R. (2014). Androgen receptor signaling in prostate cancer. Cancer Metastasis Rev. 33, 413–427. 10.1007/s10555-013-9474-0 24384911

[B11] Freund R. Lachelt U. Gruber T. Ruhle B. Wuttke S. (2018). Multifunctional efficiency: extending the concept of atom economy to functional nanomaterials. ACS Nano 12, 2094–2105. 10.1021/acsnano.8b00932 29533060

[B12] Gim H. J. Park J. Jung M. E. Houk K. N. (2021). Conformational dynamics of androgen receptors bound to agonists and antagonists. Sci. Rep. 11, 15887. 10.1038/s41598-021-94707-2 34354111 PMC8342701

[B13] Hara T. Miyazaki J. Araki H. Yamaoka M. Kanzaki N. Kusaka M. (2003). Novel mutations of androgen receptor: a possible mechanism of bicalutamide withdrawal syndrome. Cancer Res. 63, 149–153. 12517791

[B14] Horoszewicz J. S. Leong S. S. Chu T. M. Wajsman Z. L. Friedman M. Papsidero L. (1980). The LNCaP cell line--a new model for studies on human prostatic carcinoma. Prog. Clin. Biol. Res. 37, 115–132. 7384082

[B15] Hussain M. Saad F. Sternberg C. N. (2018). Enzalutamide in castration-resistant prostate cancer. N. Engl. J. Med. 379, 1381. 10.1056/NEJMc1810065 30281993

[B16] Joseph J. D. Lu N. Qian J. Sensintaffar J. Shao G. Brigham D. (2013). A clinically relevant androgen receptor mutation confers resistance to second-generation antiandrogens enzalutamide and ARN-509. Cancer Discov. 3, 1020–1029. 10.1158/2159-8290.CD-13-0226 23779130

[B17] Korpal M. Korn J. M. Gao X. Rakiec D. P. Ruddy D. A. Doshi S. (2013). An F876L mutation in androgen receptor confers genetic and phenotypic resistance to MDV3100 (enzalutamide). Cancer Discov. 3, 1030–1043. 10.1158/2159-8290.CD-13-0142 23842682

[B18] Ledet E. M. Lilly M. B. Sonpavde G. Lin E. Nussenzveig R. H. Barata P. C. (2020). Comprehensive analysis of *AR* alterations in circulating tumor DNA from patients with advanced prostate cancer. Oncol. 25, 327–333. 10.1634/theoncologist.2019-0115 32297439 PMC7160408

[B19] Lee-Ruff E. Mladenova G. (2003). Enantiomerically pure cyclobutane derivatives and their use in organic synthesis. Chem. Rev. 103, 1449–1483. 10.1021/cr010013a 12683788

[B20] Liu H. An X. Li S. Wang Y. Li J. Liu H. (2015). Interaction mechanism exploration of R-bicalutamide/S-1 with WT/W741L AR using molecular dynamics simulations. Mol. Biosyst. 11, 3347–3354. 10.1039/c5mb00499c 26442831

[B21] Liu H. Han R. Li J. Liu H. Zheng L. (2016). Molecular mechanism of R-bicalutamide switching from androgen receptor antagonist to agonist induced by amino acid mutations using molecular dynamics simulations and free energy calculation. J. Computer-Aided Mol. Des. 30, 1189–1200. 10.1007/s10822-016-9992-2 27848066

[B22] Liu H. Wang L. Tian J. Li J. Liu H. (2017a). Molecular dynamics studies on the enzalutamide resistance mechanisms induced by androgen receptor mutations. J. Cell. Biochem. 118, 2792–2801. 10.1002/jcb.25928 28181296

[B23] Liu H.-L. Zhong H.-Y. Song T.-Q. Li J.-Z. (2017b). A molecular modeling study of the hydroxyflutamide resistance mechanism induced by androgen receptor mutations. Int. J. Mol. Sci. 18, 1823. 10.3390/ijms18091823 28832499 PMC5618476

[B24] Nangia A. K. Desiraju G. R. (2019). Crystal engineering: an outlook for the future. Angew. Chem. Int. Ed. 58, 4100–4107. 10.1002/anie.201811313 30488598

[B25] Pollock J. A. Wardell S. E. Parent A. A. Stagg D. B. Ellison S. J. Alley H. M. (2016). Inhibiting androgen receptor nuclear entry in castration-resistant prostate cancer. Nat. Chem. Biol. 12, 795–801. 10.1038/nchembio.2131 27501397 PMC5030124

[B26] Schomaker J. M. Delia T. J. (2001). Arylation of halogenated pyrimidines via a Suzuki coupling reaction. J. Org. Chem. 66, 7125–7128. 10.1021/jo010573+ 11597240

[B27] Sinnwell M. A. Groeneman R. H. Ingenthron B. J. Li C. Macgillivray L. R. (2021). Supramolecular construction of a cyclobutane ring system with four different substituents in the solid state. Commun. Chem. 4, 60. 10.1038/s42004-021-00493-3 36697559 PMC9814370

[B28] Steketee K. Timmerman L. Ziel‐Van Der Made A. C. J. Doesburg P. Brinkmann A. O. Trapman J. (2002). Broadened ligand responsiveness of androgen receptor mutants obtained by random amino acid substitution of H874 and mutation hot spot T877 in prostate cancer. Int. J. Cancer 100, 309–317. 10.1002/ijc.10495 12115546

[B29] Tan J. Q. Chang J. H. Deng M. Z. (2004). 2004. - the facile route to stereodefined alkenylsubstituted pyrimidines. Synth. Commun. 34, 3773–3783. 10.1081/scc-200032495

[B30] Tran C. Ouk S. Clegg N. J. Chen Y. Watson P. A. Arora V. (2009). Development of a second-generation antiandrogen for treatment of advanced prostate cancer. Science 324, 787–790. 10.1126/science.1168175 19359544 PMC2981508

[B31] Van Der Kolk M. R. Janssen M. A. C. H. Rutjes F. P. J. T. Blanco‐Ania D. (2022). Cyclobutanes in small‐molecule drug candidates. ChemMedChem 17, e202200020. 10.1002/cmdc.202200020 35263505 PMC9314592

[B32] Veldscholte J. Ris-Stalpers C. Kuiper G. G. Jenster G. Berrevoets C. Claassen E. (1990). A mutation in the ligand binding domain of the androgen receptor of human LNCaP cells affects steroid binding characteristics and response to anti-androgens. Biochem. Biophys. Res. Commun. 173, 534–540. 10.1016/s0006-291x(05)80067-1 2260966

[B33] Xu Y. Conner M. L. Brown M. K. (2015). Cyclobutane and cyclobutene synthesis: catalytic enantioselective [2+2] cycloadditions. Angew. Chem. Int. Ed. 54, 11918–11928. 10.1002/anie.201502815 26333192

