# Supplementary material for: Profiling crystal engineered ligands for targeting treatment resistant androgen receptors
Source: Front Pharmacol. 2026 Sep 28;17:1813002. doi: 10.3389/fphar.2026.1813002 (PMC13655391; doi:10.3389/fphar.2026.1813002)
Supplement: Supplementary file 1 [file Supplementaryfile1.docx]

Supplementary Material

# 1 Supplementary Methods

**Supplementary methods**

General Synthetic Methods. All reagents were used as purchased. Tetrahydrofuran, 1,4-Dioxane, diethyl ether, Dichloromethane, and Ethyl acetate used in reactions were dried anhydrous. Solvents used for extraction and flash chromatography were reagent grade, purchased from Fisher Scientific. All reactions were carried out under dry N_2_ atmosphere except where noted. The organic products were extracted with EtOAc and washed with brine dried over Na_2_SO_4_ and concentrated under reduced pressure. The product was purified using 10% ethyl acetate in hexanes to yield pure solids. ^1^H and ^13^C NMR spectra were obtained on 400 MHz Bruker NMR spectrometers. High resolution mass spectra were obtained using electrospray ionization on a Micromass Q-Tof Ultima, and Powder X-ray diffraction (PXRD) data were collected on a Bruker D8 Advance X-ray diffractometer using CuKα1 radiation (λ = 1.5418 Å) in the range 5–45°.

**Synthesis of (E)-2-Bromo-4-styrylpyrimidine.**

According to the coupling reaction described by Tan et al (Tan et al., 2004). 1 trans-2-phenylvinylboronic acid (0.8226 g, 1 eq, 5.6 mmol) was dissolved in 25 mL THF. The catalyst, PdCl_2_(PPh_3_)_2_ (117 mg, 0.03 eq, 0.17 mmol) was added, then potassium phosphate tribasic (2.377 g, 2 eq, 5.6 mmol) was also added. The 2,4-dibromopyrimidine (1.332 g, 1 eq, 5.6 mmol) was dissolved in 5 mL THF and then added to the stirred solution. Water (5 mL) was added. The flask was equipped with a condenser and brought to a reflux for 24 hours. The reaction was quenched with 25 mL water. The organic products were extracted with 3X25 mL EtOAc. They were mixed and washed with brine (30 mL), dried over Na_2_SO_4_ and concentrated under reduced pressure. The product was purified using 10% ethyl acetate in hexanes to yield pure 0.8062 g (61%) of off-white solid.

^1^H NMR (400 MHz, DMSO) δ 8.97 (s, 2H), 7.95 (d, J = 16.0 Hz, 1H), 7.80 – 7.73 (m, 2H), 7.49 – 7.32 (m, 4H), 7.26 (d, J = 16.1 Hz, 1H).

^13^C NMR (101 MHz, DMSO) δ 165.24, 160.66, 138.63, 135.05, 129.97, 128.92, 128.06, 124.59, 118.24.

HRMS (ESI^+^) *m/z* calculated for C_12_H_9_BrN_2_^+^ 261.0022, found 261.0024.

The synthesis and characterization of (E)-2-chloro-4-styrylpyrimidine and ClCB has been reported as Compound 10 (Pollock et al., 2016).

**Solid state Irradiation of (E)-2-Bromo-4-styrylpyrimidine.**

Finely ground samples of (E)-2-Bromo-4-styrylpyrimidine (200 mg) were immersed between two Pyrex glass plates and irradiated using a 500 W broadband mercury lamp. ^1^H NMR spectroscopy was used to monitor the progress of the reaction, and near 100% conversion was achieved in 12 h. The photoproducts were purified through recrystallization using 10% ethyl acetate in hexanes to yield pure light brown solids in a near quantitative yield.

^1^H NMR (400 MHz, DMSO) δ 8.38 (d, J = 5.1 Hz, 2H), 7.37 (d, J = 5.1 Hz, 3H), 7.27 – 7.15 (m, 10H), 7.15 – 7.02 (m, 4H), 4.88 – 4.75 (m, 2H), 4.76 – 4.63 (m, 2H).

^13^C NMR (400 MHz, DMSO) δ 172.04, 159.28, 151.87, 138.45, 127.98, 127.81, 126.48, 120.54, 46.63, 43.92.

HRMS (ESI^+^) *m/z* calculated for C_24_H_18_Br_2_N_4_^+^ 542.9790, found 540.9780.

**Solubilities:** Both **ClCB** and **BrCB** are soluble in most organic solvents including ethanol, methanol, DMSO, chloroform, ethyl acetate, and acetone. The compounds are slightly soluble in benzene and hexanes.

# 2 Supplementary Figures and Tables

## 2.1 Supplementary Figures

**Supplementary Figure 1.** Synthesis scheme for **ClCB** and **BrCB**.

**Supplementary Figure 2.** ^1^H NMR (400 MHz, DMSO) of (*E*)-2-Bromo-4-styrylpyrimidine

**Supplementary Figure 3.** ^13^C NMR (400 MHz, DMSO) of (*E*)-2-Bromo-4-styrylpyrimidine

**Supplementary Figure 4.** HRMS (ESI^+^) spectrum of **BrSP**

**Supplementary Figure 5.** ^1^H NMR (400 MHz, DMSO) of **BrCB**

**Supplementary Figure 6.** ^13^C NMR (400 MHz, DMSO) of **BrCB**

**Supplementary Figure 7.** HRMS (ESI^+^) spectrum of **BrCB**

**Supplementary Figure 8.** ^1^H NMR (400 MHz, DMSO) of (*E*)-2-Chloro-4-styrylpyrimidine

**Supplementary Figure 9.** ^13^C NMR (400 MHz, DMSO) of (*E*)-2-Chloro-4-styrylpyrimidine

**Supplementary Figure 10.** ^1^H NMR (400 MHz, DMSO) of **ClCB**

**Supplementary Figure 11.** ^13^C NMR (400 MHz, DMSO) of **ClCB**

**Supplementary Figure 12.** PXRD diffractogram of **BrSP**

**Supplementary Figure 13.** PXRD diffractogram of **BrCB**

**Supplementary Figure 14.** PXRD diffractogram of **ClSP**

**Supplementary Figure 15.** PXRD diffractogram of **ClCB**

**Supplementary Figure 16.** Crystal structures ClSP and ClCB. **(A)** head-to-tail dimer showing the stacking of the C=C bonds of the alkene with 3.33 Å distance **(B)** 1D edge-to-edge view of sheets encompassing neighboring molecules of **ClSP** supported by C-H‧‧‧N hydrogen bond and **(C)** 2D C-H‧‧‧N intermolecular interactions **(D)** Cl‧‧‧Cl halogen bond of the nearest neighbor **ClSP** (green) **(E)** Single crystal structure for **ClCB** highlighting the *rctt* isomer **(F)** Extended packing of the **ClCB** showing the interaction of the nearest neighbor to form a planar sheet. Corresponding color identifiers for atoms include hydrogen (white), carbon (grey), nitrogen (blue) and chlorine (green). All intermolecular interactions are indicated by dotted blue lines

**Supplementary Figure 17.** Validation experiments on the transcriptional response of WT AR and AR^F877L/T878A^ to traditional AAs. (A) The LNCaP/AR cell line is susceptible to inhibition by flutamide due to codon switch induced to reverse the A878 residue back to T878. qPCR quantification of gene expression in WT AR cells after 24hr exposure to 1nM R1881 or 1nM R1881 + 20µM AAs. Notably, flutamide reduces *KLK3* and *NKX3.1* expression with similar ability to second generation anti-androgens. (B) Enzalutamide loses antagonistic function in the MR49F cell line due to the F877L point mutation. qPCR quantification of gene expression in MR49F cells after 24hr exposure to 1nM R1881 or 1nM R1881 + 20µM AAs. Enza is unable to block the expression of *KLK3* and *NKX3.1* after exposure to synthetic androgen indicating therapeutic resistance.

**Supplementary Figure 18.** Evaluation of agonistic, partial agonistic, or antagonistic activity of AAs and CBs in LNCaP cells. (A) Representative ICC images of AR (red) and changes in protein localization post 4hr treatment with compounds. (B and C) Quantification of AR protein colocalization with the nuclear marker DAPI (blue) by Manders 2 coefficient and representation of the nuclear/cytoplasmic fraction of AR protein. Studies were performed after hormone deprivation to evaluate the presence of agonistic, partial agonistic, or antagonistic activity of each compound in the absence of androgen.

**Supplementary Figure 19.** Evaluation of agonistic, partial agonistic, or antagonistic activity of AAs and CBs in MR49F cells. (A) Representative ICC images of AR (red) and changes in protein localization post 4hr treatment with compounds. (B and C) Quantification of AR protein colocalization with the nuclear marker DAPI (blue) by Manders 2 coefficient and representation of the nuclear/cytoplasmic fraction of AR protein. Studies were performed after hormone deprivation to evaluate the presence of agonistic, partial agonistic, or antagonistic activity of each compound in the absence of androgen.

**Supplementary Figure 20.** 2D interaction diagram of receptor-ligand complex. 2D representation of ClCB (left) and BrCB (right) within the (A, B) WT, (C, D) T877A mutant, and (E, F) F877L/T878A double mutant AR LBD respectively, obtained by docking. The legend interpretation of the ligand-receptor complex interactions is listed at the bottom of the figure. The directional arrows indicate the acceptor/donor nature. The proximity contour represented as a dotted outline that surrounds the ligand, is proportional to the proximity of the ligand to the receptor cavity.

**Supplementary Table 1**. Crystallographic data and structure refinement parameters of ClSP, [(E)-2-Bromo-4-styrylpyrimidine] (BrSP), [(E)-2-Chloro-4-styrylpyrimidine]

| **Identification code** | **BrSP** | **ClSP** | **BrCB** | **ClCB** |
| --- | --- | --- | --- | --- |
| **CCDC deposition number** | 2484979 | 2484980 | 2484981 | 2484982 |
| **Empirical formula** | C_12_H_9_BrN_2_ | C_12_H_9_ClN_2_ | C24H18Br2N4 | C24H18Cl2N4 |
| **Formular weight g/mol** | 261.12 | 216.67 | 522.24 | 433.33 |
| **Temperature/K** | 100.0 | 100.0 | 173.0 | 100.0 |
| **Crystal system** | monoclinic | monoclinic | triclinic | triclinic |
| **Space group** | P2(1)/n | P2(1)/n | P-1 | P-1 |
| **a/Å** | 7.336 (6) | 7.280 (3) | 9.731 (3) | 9.575 (4) |
| **b/Å** | 12.599 (4) | 12.498 (3) | 11.301 (4) | 11.338 (4) |
| **c/Å** | 11.391 (3) | 11.279 (6) | 11.517 (4) | 11.415 (4) |
| **α/°** | 90 | 90 | 109.663 (7) | 111.035 (7) |
| **β/°** | 98.485 | 98.701 (3) | 92.543 (8) | 90.699 (7) |
| **γ/°** | 90 | 90 | 111.258 (7) | 114.424 (9) |
| **Volume/Å^3^** | 1041.4 (5) | 1014.4 (11) | 1062.5 (6) | 1034(10) |
| **Z** | 4 | 4 | 2 | 2 |
| **ρ_calc_ (g/cm^3^)** | 1.666 | 1.419 | 1.362 | 1.392 |
| **μ/mm^‑1^** | 3.911 | 0.339 | 0.103 | 0.333 |
| **F(000)** | 520.0 | 448.0 | 520.0 | 448.0 |
| **Crystal size/mm^3^** | 0.095 x 0.198 x 0.297 | 0.033 x 0.090 x 0.253 | 0.080 x 0.190 x 0.312 | 0.156 x 0.202 x 0.243 |
| **Goodness-of-fit on F^2^** | 1.099 | 1.027 | 1.098 | 1.061 |

**Table S2a**. Interaction report for the ligand **ClCB** in complex with the WT AR LBD.

| **WT AR LBD** | | **Type of interaction** | **Distance**  **(Å)** | **E (kcal/mol)** |
| --- | --- | --- | --- | --- |
| **Atom** | Residue |  |  |  |
| **SD** | MET742 | H-bond | 3.00 | -0.5 |
| **SD** | MET787 | H-bond | 2.64 | -0.5 |

**Table S2b**. Interaction report for the ligand **BrCB** in complex with the T877A mutant AR LBD.

| **T877A mutant AR LBD** | | **Type of interaction** | **Distance**  **(Å)** | **E (kcal/mol)** |
| --- | --- | --- | --- | --- |
| **Atom** | Residue |  |  |  |
| **-** | TRP741 | Aromatic | 4.45 | - |
| **SD** | CYS784 | H-bond | 3.48 | -0.5 |

**Table S2c.** Interaction report for the ligand **ClCB** in complex with the F877L/T878A double mutant AR LBD.

| **F877L/T878A double mutant AR LBD** | | **Type of interaction** | **Distance**  **(Å)** | **E (kcal/mol)** |
| --- | --- | --- | --- | --- |
| **Atom** | Residue |  |  |  |
| **SD** | MET788 | H-bond | 2.46 | -0.8 |

**Table S2d**. Interaction report for the ligand **BrCB** in complex with the F877L/T878A double mutant AR LBD.

| **F877L/T878A double mutant AR LBD** | | **Type of interaction** | **Distance**  **(Å)** | **E (kcal/mol)** |
| --- | --- | --- | --- | --- |
| **Atom** | Residue |  |  |  |
| **OE1** | GLN712 | Halogen bond | 3.79 | -1.8 |

**Supplemental References**

POLLOCK, J. A., WARDELL, S. E., PARENT, A. A., STAGG, D. B., ELLISON, S. J., ALLEY, H. M., CHAO, C. A., LAWRENCE, S. A., STICE, J. P., SPASOJEVIC, I., BAKER, J. G., KIM, S. H., MCDONNELL, D. P., KATZENELLENBOGEN, J. A. & NORRIS, J. D. 2016. Inhibiting androgen receptor nuclear entry in castration-resistant prostate cancer. *Nature Chemical Biology,* 12**,** 795-801.

TAN, J. Q., CHANG, J. H. & DENG, M. Z. 2004. The facile route to stereodefined alkenyl-substituted pyrimidines. *Synthetic Communications,* 34**,** 3773-3783.
